# The use of proteomics in identifying differentially expressed serum proteins in humans with type 2 diabetes

**DOI:** 10.1186/1477-5956-4-22

**Published:** 2006-12-12

**Authors:** Tea Sundsten, Michael Eberhardson, Michael Göransson, Peter Bergsten

**Affiliations:** 1 Department of Medical Cell Biology, Uppsala University, Uppsala, Sweden; 2 Department of Medicine, Karolinska Institutet, Stockholm South Hospital, Stockholm; 3 Enköping Hospital, Enköping, Sweden

## Abstract

**Background:**

The aim of the study was to optimize protocols for finding and identifying serum proteins that are differentially expressed in persons with normal glucose tolerance (NGT) compared to individuals with type 2 diabetes mellitus (T2DM).

Serum from persons with NGT and persons with T2DM was profiled using ProteinChip arrays and time-of-flight mass spectra were generated by surface enhanced laser desorption/ionization time-of-flight mass spectrometry (SELDI-TOF MS).

**Results:**

Mass spectra from NGT- and T2DM-groups were compared. Fifteen proteins ranging from 5 to 79 kDa were differentially expressed (p < 0.05). Five of these proteins showed decreased and ten showed increased serum levels in individuals with T2DM. To be able to identify the proteins, the complexity of the sample was reduced by fractionation approaches. Subsequently, the purified fractions containing biomarkers were separated by one-dimensional SDS-polyacrylamide gel electrophoresis (SDS-PAGE) in two identical lanes. Protein bands of the first lane were excised and subjected to passive elution to recapture the biomarkers on ProteinChip arrays. The corresponding bands of the second lane were subjected to peptide-mass fingerprinting (PMF). Using this approach four of the differentially expressed proteins were identified as apolipoprotein C3 (9.4 kDa), transthyretin (13.9 kDa), albumin (66 kDa) and transferrin (79 kDa). Whereas apolipoprotein C3 and transthyretin were up-regulated, albumin and transferrin were down-regulated in T2DM.

**Conclusion:**

Protocols for protein profiling by SELDI-TOF MS and protein identification by fractionation, SDS-PAGE and PMF were optimized for serum from humans with T2DM. With these protocols differentially expressed proteins were discovered and identified when serum from NGT- and T2DM-individuals was analyzed.

## Background

Elevated fasting glucose levels and extended postprandial hyperglycemia are cardinal traits of individuals with type 2 diabetes mellitus (T2DM) [1-3]. Impaired early insulin secretory response of the pancreatic β-cell and decreased insulin sensitivity of muscle, fat and liver cells contribute to the state of hyperglycemia [3-5]. Prolonged hyperglycemia is associated with both micro- and macrovascular complications, which are leading causes of morbidity and death in diabetes [6-9]. With increasing numbers of persons demonstrating T2DM much attention has been paid to finding causes for the development of deranged glucose homeostasis. Hypotheses implicating circulating proteins in the pathogenesis of T2DM have been tested by measuring levels of these proteins in serum obtained from persons with different degrees of glucose intolerance. In this way the role of specific proteins in the development of T2DM has been investigated with TNF-α, interleukin 6 and adiponectin as recent examples [10,11]. However, T2DM is a complex disease with altered expression of many genes and their products [12,13], which can be anticipated to interact. It is therefore critical to monitor changes in many proteins simultaneously. Our first aim with this study was to optimize a protocol for measuring levels of several proteins simultaneously, which were generated from serum samples obtained from persons with normal glucose tolerance (NGT) or T2DM by surface enhanced laser desorption/ionization time-of-flight mass spectrometry (SELDI-TOF MS) [14]. With this proteomic approach differentially expressed proteins or biomarkers in individuals with NGT compared to T2DM were discovered. The second aim was to develop a protocol for identification of these biomarkers. Crude serum contains thousands of proteins [15], which made it necessary to reduce the sample complexity. This was achieved by fractionating the serum with anionic columns followed by either size fractionation or reverse phase beads. Finally, the proteins were separated by one-dimensional SDS-polyacrylamide gel electrophoresis (SDS-PAGE). The identities of the differentially expressed serum proteins were obtained by peptide mass fingerprinting (PMF).

## Results

### Study subjects and their categorization

The study contained six participants, two women and four men (Table 1). Fasting plasma glucose (FPG) concentrations were obtained from the study subjects after an over-night fast. After intake of glucose the plasma glucose concentration was measured at 120 min (G_120_). Using the WHO classification [16] the subjects were categorized as having NGT or T2DM. Two of the T2DM persons were diet-treated and one was drug-treated (Subject 6).

**Table 1:** Subject characterization and categorization

Subject	Gender	Age	BMI	Fasting glucose	G_120_	WHO Category
		years	kg/m^2^	(mM)	(mM)	

1	Female	57	20.6	4.5	5.9	NGT
2	Male	65	26.3	5.5	4.9	NGT
3	Male	57	21.9	5.9	3.4	NGT
4	Male	54	30.7	7.3	7.8	T2DM
5	Male	56	26.6	8.8	10.9	T2DM
6	Female	54	23.1	14.1	21.9	T2DM

Subjects were ranked based on fasting plasma glucose concentration. The plasma glucose concentrations at 0 min (fasting) and 120 min (G_120_) after an oral glucose tolerance test were used to categorize the subjects to have normal glucose tolerance (NGT) or type 2 diabetes mellitus (T2DM). Gender, age and body mass index (BMI) are also listed.

### Finding and identifying biomarkers

Protein measurements were performed on denaturated, diluted serum samples on metal capture (IMAC30) and cationic exchanger (CM10) surfaces. Protein profiles from each subject were obtained with SELDI-TOF MS.

With the IMAC30 array in total 14, 15 and 25 peaks were found in the low, intermediate and high mass range, respectively. Out of these peaks, 12 with masses 5915, 6095, 7778, 8944, 9133, 9303, 9505, 17294, 28166, 37887, 51259 and 66355 Da were differentially expressed between NGT- and T2DM-persons (p < 0.05). The amounts of these proteins were calculated for each subject by measuring the height of each peak (peak intensity). Out of these 12 biomarkers, eight were up-regulated and four down-regulated in T2DM (Table 2).

**Table 2:** Differentially expressed proteins in serum from normal glucose tolerant and type 2 diabetes mellitus individuals

**Peak (Da)**	**Array**	**T2DM vs. NGT**
**5915**	IMAC30	↑
**6095**	IMAC30	↑
**7778**	IMAC30	↑
**8944/8949**	IMAC30/CM10	↑
**9133**	IMAC30	↑
**9303/9305**	IMAC30/CM10	↑
**9443**	CM10	↑
**9505**	IMAC30	↑
**13923**	CM10	↑
**17294**	IMAC30	↓
**28166**	IMAC30	↓
**37887**	IMAC30	↑
**51259**	IMAC30	↓
**66355/66267**	IMAC30/CM10	↓
**79040**	CM10	↓

Amounts of serum proteins were determined by calculating heights of peaks found in SELDI-TOF mass spectra using the immobilized metal affinity capture (IMAC30) and cationic exchanger (CM10) arrays. Serum samples were obtained from three normal glucose tolerant (NGT) and three type 2 diabetes mellitus (T2DM) subjects. Protein masses, after applying inclusion criteria (signal to noise ratio > 10, percentage of peaks > 20%) on the spectra, are indicated. Significant (p < 0.05, Mann-Whitney U-test) up- (↑) and down (↓) regulations are noted.

**Figure 1:**
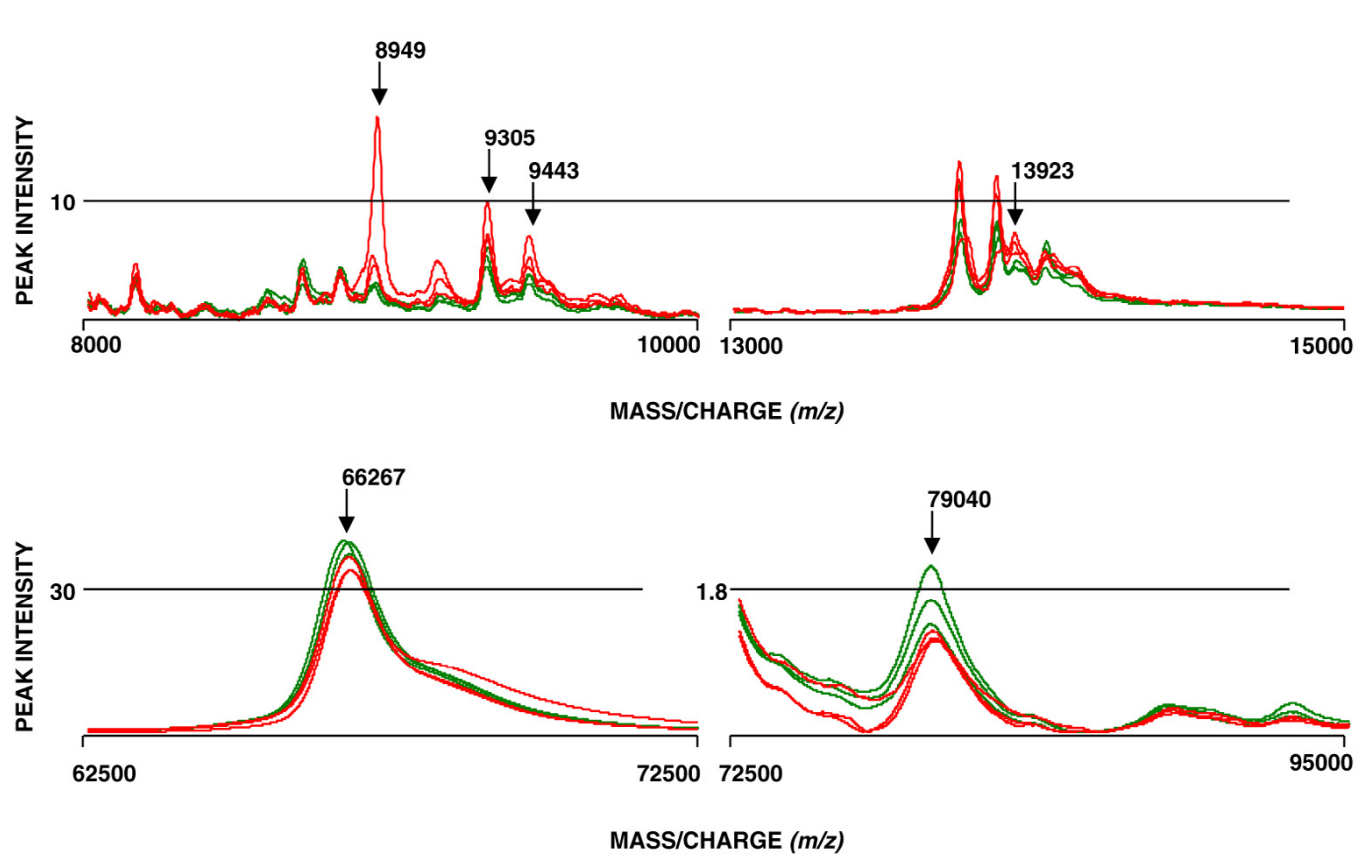
**Mass spectra showing the differentially expressed proteins**. SELDI-TOF mass spectra showing the six differentially expressed serum proteins captured by cationic exchanger array. The green spectra are from individuals with normal glucose tolerance and the red spectra are from individuals with type 2 diabetes mellitus.

With the CM10 array in total 7, 10 and 21 peaks were found in the low, intermediate and high mass range, respectively. Out of these peaks, six with masses 8949, 9305, 9443, 13923, 66267 and 79040 Da were differentially expressed between NGT- and T2DM-persons (p < 0.05). The amounts of these proteins were calculated for each subject by measuring the height of each peak (peak intensity). Out of the six biomarkers, four were up-regulated and two down-regulated in T2DM (Figure 1, Table 2).

Three of the differentially expressed proteins (8944/8949, 9303/9305 and 66355/66267 Da) were captured on both types of arrays. Using both the arrays, in total 15 differentially expressed proteins were found. Five of these proteins showed decreased levels and ten showed increased serum levels in individuals with T2DM (Table 2).

The strategy of the identification work will be exemplified by the elucidation of the identity of the 9.4 kDa biomarker. When differentially expressed serum proteins are to be identified, the complexity of the serum has to be reduced. Such reduction was achieved by fractionating denaturated serum using anionic spin columns. The different fractions were applied on normal phase (NP20) and CM10 arrays, and analyzed by SELDI-TOF MS. The latter surface was used for the identification work since focus was on the differentially expressed proteins discovered by CM10.

The flow through fraction of the anionic spin column had retained biomarkers with masses 8949, 9305 and 79040 Da. Further purification was achieved by size fractionation. The two smaller proteins easily passed through the size column, while the larger protein remained in the retentate. The retentate was subsequently re-suspended in 60 % acetonitrile (ACN).

**Figure 2:**
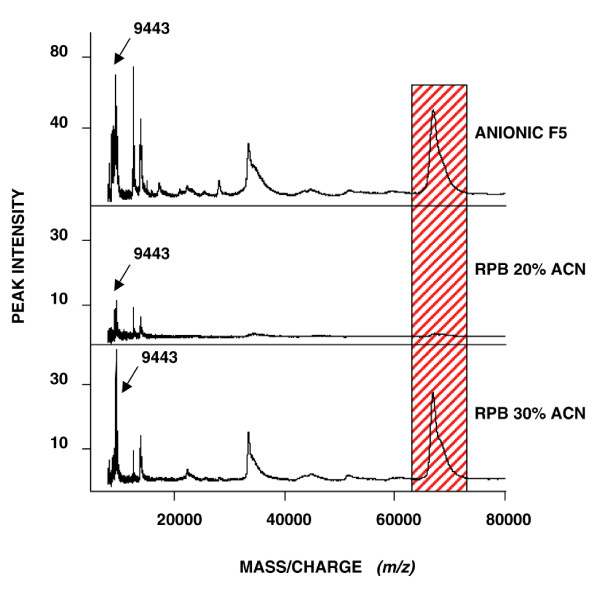
**Mass spectra of serum fractions showing the 9443 Da biomarker**. SELDI-TOF mass spectra of serum after fractionation. Upper trace shows spectrum of the anionic fraction 5. Middle trace shows spectrum of the reverse phase beads fraction with 20 % acetonitrile. Lower trace shows the reverse phase beads fraction with 30 % acetonitrile. The abundant 66 kDa protein (albumin) was reduced in reverse phase fraction with 20 % acetonitrile (hatched area). The arrow indicates the 9443 Da biomarker.

The anionic fraction 5 (pH 3) contained the biomarkers with masses 9305, 9443, 13923 and 66267 Da (Figure 2). This fraction was further purified using reverse phase beads. The 9443 and 13923 Da proteins were quite pure in both the 20 % ACN and the 30 % ACN fractions. The amount of the 66267 Da protein was quite low in the 20 % ACN fraction but higher in the 30 % ACN fraction (Figure 2).

**Figure 3:**
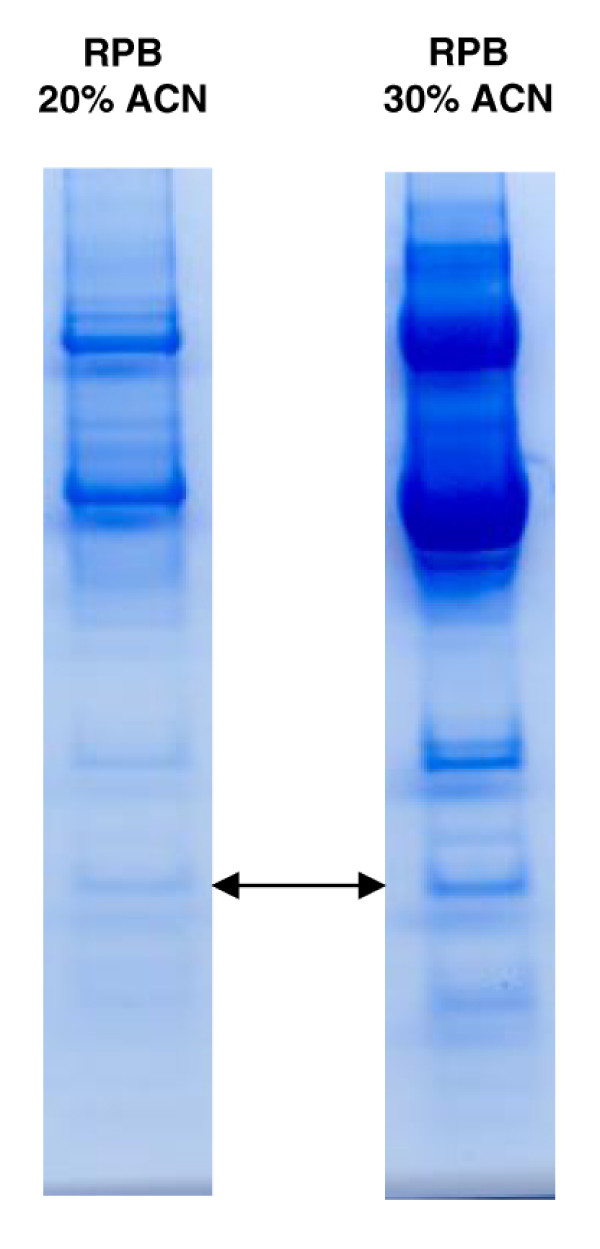
**SDS-PAGE of serum fractions**. Proteins of serum fractions containing biomarkers were separated with one-dimensional gel electrophoresis. The two reverse phase beads (RPB) fractions containing 20 and 30 % acetonitrile are shown after staining with colloidal coomassie. Bands in the relevant mass range were excised and eluted. The arrows indicate the bands containing the 9443 biomarker.

**Figure 4:**
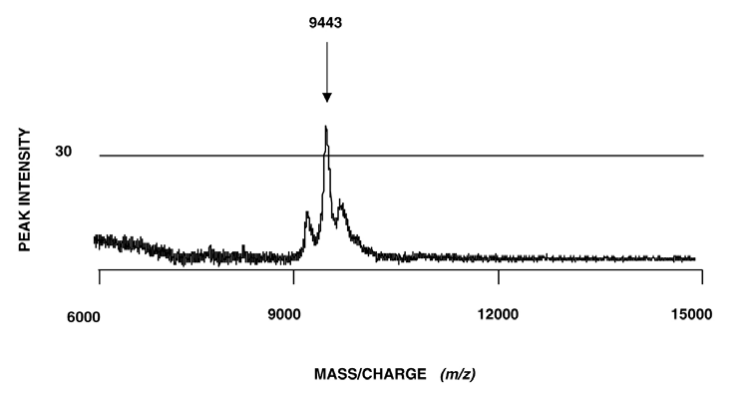
**Mass spectrum verifying the 9443 Da biomarker**. Eluates were re-applied on the arrays to confirm that the eluted protein was the discovered biomarker at 9443 Da.

The two reverse phase fractions containing the biomarkers were speed-vacuumed, resuspended in sample buffer and run in duplicates, parallel lanes on the gels. After staining, gel bands in the relevant mass range were cut out and proteins passively eluted. One lane of each reverse phase fraction is shown in Figure 3. The eluates were applied on NP20 arrays to confirm that the eluted proteins were identical to the initially discovered biomarkers (Figure 4). When the mass spectral peak of the eluted protein coincided with the biomarker peak of the serum mass spectrum, the corresponding band from the parallel lane was excised and subjected to PMF and identified using MALDI-TOF MS. Using this approach the 9443 Da protein was identified as apolipoprotein C3 (apo C3), the 13923 Da protein as transthyretin (TTR), the 66267 Da protein as albumin and the 79040 Da protein as transferrin.

## Discussion

Profiling of blood proteins by SELDI-TOF MS has been performed on blood obtained from individuals with different diseases e.g. breast or prostate cancer [17,18]. The motivation of these studies has, at least in part, been the lack of reliable diagnostic and prognostic biomarkers of the disease states. Through large patient cohort studies, patterns of differentially expressed serum proteins have been discovered that with high accuracy distinguish afflicted from healthy individuals [17]. Since such patterns are useful in themselves for diagnosis without knowing the identities of the differentially expressed proteins of the pattern, often only mass/charge of these proteins have been published [19]. In T2DM the situation is different since the blood glucose level is a reliable and easy to measure marker of the diabetes disease state. In diabetes research the SELDI-technology will probably therefore not be used so much for diagnostic and prognostic purposes. In contrast, the proteomic platform is highly relevant as a tool for the investigation of the pathophysiology of the disease since T2DM is polygenic [20] and levels of many blood-borne proteins can be expected to be altered. With this background we wanted to evaluate the usefulness of SELDI-TOF MS in investigating changes in expression patterns of blood proteins of individuals with T2DM. The aim was to see whether differentially expressed proteins could not only be discovered but also identified. In the context of type 2 diabetes the SELDI-platform has been used in a few animal studies, where it has either been used to obtain protein patterns without identifying the differentially expressed proteins [21-23] or to measure quantities of already identified proteins [24-26].

The critical part of the SELDI-platform is the ProteinChip array. The array is the surface, which adsorbs proteins of the sample applied to the surface. The surface chemistry of the array decides what subset of proteins will be adsorbed. We found the cationic exchanger and immobilized metal affinity capture surfaces to be equally efficient to retain proteins. These experiences are in line with those of other studies, where serum or plasma protein profiling has been conducted [17,18,21-23,25,26]. Important aspects, when defining the efficiency of a given chromatographic surface to generate mass spectra showing numerous proteins, are which energy-absorbing molecule and parameter settings of the mass spectrometer are used. In the present study we used sinapinic acid (SPA) as energy absorbing molecule. Furthermore, we used different laser energies for low, intermediate and high molecular weight proteins. The generated spectra were analyzed in two consecutive cycles. With the settings used in this study the number of proteins recognized in the CM10 and IMAC30 arrays was 38 and 44, respectively. More than 90 % of the peaks were detected in the first cycle. The stringency of the settings affects directly the number of peaks detected, which can be increased by lowering the signal to noise ratio (S/N). Among the discovered proteins, six proteins detected by the CM10 array and 12 proteins detected by the IMAC30 array were differentially expressed. The fact that peaks were recognized with such accuracy and determined as differentially expressed relied on the reproducibility and quantitativeness of the method, respectively. These aspects were separately addressed and verified. Whereas three differentially expressed proteins were captured by both arrays, the remaining proteins were either detected by the CM10 or IMAC30 array. These results illustrate the usefulness of employing different arrays and binding conditions, which will adsorb different subsets of proteins, and that some proteins are common to the different subsets especially when arrays have similar surface chemistries.

An important aspect of the present work was to evaluate how readily the differentially expressed proteins were identified. The strategy was to fractionate the sample to reduce its complexity. Proteins of the fractions were separated by SDS-PAGE, trypsin digested and identified by PMF. Similar strategies for proteomic profiling and identification have been used for skeletal muscle proteins [27]. When fractionating the crude serum sample, the existence of the protein of interest, the biomarker, was verified by applying the fraction on NP20 array and the array on which it was discovered. Fractions containing the biomarker, reasonably well separated from other proteins, were subjected to SDS-PAGE. By passively eluting bands thought to contain biomarkers and re-applying the eluates on NP20 arrays, the presence of the biomarker in a given band could be verified by SELDI-TOF MS. When the presence of the biomarker in the gel piece was confirmed, a similarly obtained gel piece was subjected to trypsin digestion and the identity of the protein obtained by PMF.

We chose to identify the six differentially expressed proteins obtained with the CM10 array. This choice was based on the fact that they would address at least two aspects of protein identification work. The first aspect was that many of the proteins were obscured by the 66 kDa protein, which by its abundance was assumed and later verified to be albumin, and needed to be separated from it. The second aspect was that the 8.9, 9.3 and 9.4 kDa biomarkers were aggregated. In addition, other proteins of similar mass/charge were also present in the same mass spectral area and different fractionation principles would be required. We were able to obtain identities of four of the six proteins. Although the possible biological significance of these changes of the proteins in T2DM will be discussed briefly below, it should be pointed out that differential expression was based on the comparison of three control individuals with three individuals with T2DM. With these very low numbers, the study is more intended as a case report and evaluation of the SELDI-platform for diabetes work using blood samples than having implications for the understanding of the pathophysiology of the disease.

Lowering of transferrin and albumin are characteristic changes in inflammation [28], which may also be part of the pathogenesis of T2DM [29]. Decreased circulating levels of these proteins in T2DM have also been connected with impaired kidney function [30]. However, the three T2DM individuals in this study did neither have macro- nor microalbuminurea. Increased urinary excretion of both transferrin and albumin has, nevertheless, been observed in normoalbuminuric patients with T2DM [31], which may contribute to explain our findings. Lowered levels of transferrin have previously been shown in T2DM [32].

Increased levels of apo C3 have been observed in T2DM and considered a cardiovascular risk factor [33,34]. Also, increased levels of apo C3 have been shown in serum from individuals with type 1 diabetes mellitus (T1DM) [35].

Finally, TTR was up-regulated. The protein exists both in a tetrameric and the 13.9 kDa monomeric form [36]. Whereas the tetrameric form promotes β-cell function, no such effect was observed with the monomeric form [37]. In individuals with T1DM, a rise of the blood levels of the monomeric form was observed and suggested to be associated with the development of β-cell failure in T1DM [37]. Consistent with this idea, mutations in the transcription factor hepatocyte nuclear factor-1α (HNF-1α) were associated with down-regulation of the transcription of TTR in mutant HNF-1α cells [38] and in MODY3 patients [39]. In another study the levels of TTR in patients with non-insulin dependent diabetes were not affected [40] and in inflammatory states TTR has been shown to decrease [29].

## Conclusion

In conclusion, protocols have been optimized both for measuring protein patterns with SELDI-TOF MS and identifying biomarker proteins with PMF in sera from persons with T2DM. Increasing the number of study subjects may confirm the identified, differentially expressed proteins of this study as implicated in the pathophysiology of T2DM.

## Materials and methods

### Chemicals

Chemicals of analytical grade and deionized water were used. PEFAbloc^®^, ACN and trifluoroacetic acid (TFA) came from Merck (Darmstadt, Germany). CHAPS was purchased from Sigma (St. Louis, MO) and urea from Amersham Biosciences (Uppsala, Sweden). The material used for one-dimensional SDS-PAGE, including the NuPAGE^® ^Novex gels, sample buffer, running buffer, coomassie stain and SeeBlue Plus2 pre-stained standard, all came from Invitrogen (Carlsbad, CA). The ProteinChip arrays, all-in-1 peptide and all-in-1 protein molecular weight standards, Q ceramic HyperD F spin columns, reverse phase beads and SPA came from Ciphergen (Freemont, CA).

### Study subjects

The study was performed on six subjects, two women and four men. Whereas three subjects had NGT, three had T2DM. The latter subjects were treated with anti-diabetes drugs or diet. The drug treatment was discontinued two days prior to the study. The study subjects were between 54 and 65 years old, had normal blood pressure, did not have any other metabolic or cancerous disease, were non-smoking and without any medication affecting insulin secretion. All subjects had normal thyroid, liver, cardio-pulmonary and kidney function as determined by medical history, physical examination and blood chemistry screening. The study participants with T2DM were found among patients at the Diabetic unit at the Medical Center, Enköping hospital, Sweden. The control subjects were recruited by advertisement. Written informed consent was obtained from all participants. After a 75-g oral glucose tolerance test (OGTT) the study participants were categorized using the WHO criteria [16] as having NGT or T2DM. The study protocol was approved by the Human Ethics Committee at the Medical Faculty, Uppsala University, Uppsala, Sweden.

### Oral glucose tolerance test

All subjects fasted over-night (10–12 hours). At 8:30 am an intravenous catheter was placed in the antecubital vein. Blood was drawn from the catheter and plasma was prepared for glucose concentration measurements by collecting blood in EDTA-containing tubes. After centrifugation (2400 g) of the tubes, plasma was separated and the glucose concentration was measured. Serum was prepared for protein profiling measurements. In such blood samples PEFAbloc^® ^was added before the samples were allowed to clot. The serum samples were aliquoted and stored in -70°C until analysis. Samples for blood chemistry were taken and analyzed according to standard procedures. After this the subject received 75 g of glucose per os (time point 0 min) and blood samples were collected after 120 min for glucose concentration measurements. The plasma glucose concentrations were determined by the glucose oxidase reaction using Granutest 250 glucose (Merck, Darmstadt, Germany).

### Measurements of protein patterns

Serum protein patterns were determined by SELDI-TOF MS using ProteinChip arrays. Serum samples were thawn on ice, quickly vortexed and centrifuged. Samples (50 μl) were denaturated by adding 75 μl 50 mM Trizma^® ^base buffer (pH 9) containing 9 M urea and 2 % CHAPS. The samples were vortexed for 30 min at 4°C and centrifuged (12,000 g) for 5 min. Finally, the samples were diluted by mixing 5 μl denaturated serum with 245 μl binding buffer defined below.

Two types of arrays with different surface chemistries were used to capture proteins from the serum samples. IMAC30 arrays were activated by washing the spots during 5 min with 50 μl 100 mM copper(II)sulphate twice. The copper(II)sulphate solution was discarded, the arrays were placed in a Bioprocessor (Ciphergen) and briefly washed with water. Then the arrays were equilibrated during 2 × 5 min with binding buffer consisting of 100 mM sodium phosphate (pH 6) after which the denaturated, diluted serum samples (100 μl) were applied on the spots of the arrays. The Bioprocessor was sealed and left at room temperature (RT) on a shaker for 1 hour. The excess sample was then discarded and the arrays were washed three times with 125 μl sodium phosphate buffer (pH 6) during 5 min. The arrays were finally quickly washed twice with water, removed from the Bioprocessor and allowed to air dry. Saturated solution of SPA was prepared by mixing 5 mg SPA with 100 μl 100 % ACN and 100 μl 1 % TFA. The saturated SPA solution was diluted to 50 % and 2 × 1 μl was applied to every spot. The spots were air dried in between.

CM10 arrays were equilibrated during 2 × 5 min with binding buffer consisting of 100 mM acetate buffer (pH 4). The arrays were placed in the Bioprocessor, after which the denaturated, diluted serum samples (100 μl) were applied on the spots of the arrays. Binding and washing steps were performed as described for IMAC30, using acetate buffer instead of phosphate buffer.

The arrays were placed in the SELDI MS, and time-of-flight spectra were generated by averaging several laser shots at different laser intensities, depending on which mass region was studied. For the low molecular weight range (2 to 50 kDa) the following settings were used: laser intensity 220, detector sensitivity 8 and optimization range 2–10 kDa. The mass accuracy was calibrated externally using the All-in-1-peptide molecular weight standard. For the intermediate molecular weight range (5 to 70 kDa) the following settings were used: laser intensity 220, detector sensitivity 8 and optimization range 5–15 kDa. The mass accuracy was calibrated externally using the All-in-1-protein molecular weight standard. For the high molecular weight range (15 to 150 kDa) the following settings were used: laser intensity 240, detector sensitivity 8 and optimization range 15–50 kDa. The mass accuracy was calibrated externally using the All-in-1-protein molecular weight standard. Peak normalization, peak detection and alignment were performed with Ciphergen ProteinChip Software 3.1.

Biomarker discovery requires the method to be reproducible and quantitative. Reproducibility of the arrays was addressed by applying four equal aliquots of a serum sample onto spots of the arrays. The intensities of three peaks were determined for each spot. The coefficient of variance was less than 10 % for all three peaks. Quantitation of the arrays was tested by diluting a serum sample and intensities of three peaks were determined for each dilution. The peak intensities decreased as the sample was diluted and showed strong linear correlations (r^2^>0.95) when plotted against the logarithm of the dilutions.

### Analysis

Mass spectra of serum protein patterns were obtained from each subject. The ProteinChip software settings for peak detection were set at a quite rigorous level. The first pass S/N was set at 10, and the peak had to be present in 20 % of the spectra. For a given peak to be considered for comparison, the mass error had to be < 0.3 %. Peaks were selected for further analysis if their heights were significantly different when comparison was performed between the NGT- and T2DM-groups using the Mann-Whitney U-test.

### Serum protein identification

Identities of biomarkers, which are differentially displayed proteins obtained by SELDI-TOF MS, were obtained by PMF. The procedure involved sample fractionation, SDS-PAGE of fractionated serum samples, elution and verification of biomarkers, excision of relevant gel bands, in gel trypsin digestion, mass determination of the tryptic fragments and peptide database comparison. The first step in identifying the biomarkers was to reduce sample complexity by anionic fractionation using Q ceramic HyperD F spin columns. The columns were first equilibrated with a 50 mM Trizma^® ^base buffer with 0.9 M urea and 0.2 % CHAPS. Denaturated serum (250 μl) was diluted with another 250 μl of the same buffer and bound to the resin during 30 min on a mixer at RT. The column was then centrifuged (1 min, 500 g) and the flow through fraction was collected into an eppendorf tube. The column was then step-wise washed with 500 μl of buffers with pH 9, 7, 5, 4, 3 or organic buffer. The fractions were applied on both NP20 arrays and the arrays, which were used when discovering the biomarkers.

To further purify the anionic fractions, size fractionation with microcon YM-50 columns (Millipore, Billerica, MA) or hydrophobic fractionation using reverse phase beads were used. When performing the size fractionation, the sample was added to the size fractionation column and concentrated by centrifugation. With repeated washing steps using increasing percentage of ACN (10 %, 20 %, 30 %, 40 %, 50 % and 60 %) in 0.1 % TFA solution, the serum sample was further purified. These fractions were also analyzed with NP20 arrays, to locate the biomarker proteins to the different fractions.

When performing the hydrophobic fractionation, serum proteins were bound to reverse phase beads during 30 min shaking at RT. Beads were spun down by centrifugation and the supernatant was removed. Subsequently, the beads were shaken with increasing percentage of ACN (10 %, 20 %, 30 %, 40 %, 50 % and 60 %) and fractions removed between washing steps. The hydrophobic fractions were also analyzed on NP20 arrays.

Fractions containing the proteins of interest were speed-vacuumed in a concentrator (Eppendorf, Hamburg, Germany) to remove the buffer. Subsequently, the pellets were resuspended with NuPAGE^® ^sample buffer. The fractions were resolved by applying samples in parallel lanes on NuPAGE^® ^Novex 4–12 % Bis-Tris gels. Also, a SeeBlue Plus2 pre-stained standard was run on the gel. After electrophoresis, the gels were stained with colloidal coomassie stainer and scanned. From the first lane protein bands of the relevant masses were excised, cut into smaller pieces and passively eluted. The elution was achieved by placing the gel pieces into a micro tube and shaken for 30 min in 300 μl of solution containing 50 % water, 40 % methanol and 10 % ACN. Afterwards the solution was removed and the bands were washed with 200 μl of 50 % ACN in 50 mM ammonium bicarbonate buffer (pH 8). After 30 min shaking, the buffer was removed and the gel pieces dehydrated for 15 min in 200 μl 100 % ACN. Subsequently, the ACN was removed and the gel pieces were heated during 5 min in a thermo block (60°C). Approximately 30 μl of an elution buffer consisting of 45% formic acid, 30 % ACN, 10 % isopropanol and 15 % water was added to the tubes, which were vortexed briefly and left on a shaker at RT over-night. Subsequently, 1 μl of the eluent was applied on an NP20 array, to see if the biomarker protein had been eluted from the gel.

When eluates contained biomarkers, the corresponding band of the second lane was excised and de-stained. In-gel digestion was performed with trypsin essentially as described [41]. Proteins were identified by PMF with the search program Mascot (Matrix Science, London, UK). SWISS-PROT was used as the protein sequence database and the peptide masses were compared to the theoretical peptide masses of all available proteins from mammalian taxonomy. The criteria used to accept identifications included the extent of sequence coverage, number of peptides matched and molecular weight search (MOWSE) score. Information about identified protein and putative function was found at the SWISS-PROT database at the ExPASy Molecular Biology Server [42].

## Competing interests

The author(s) declare that they have no competing interests.

## Authors' contributions

All authors designed the study. TS, ME and MG were involved in the sample collection. TS carried out all the SELDI-TOF experiments. TS and PB evaluated the results and drafted the manuscript.
